# Change in Cav3.2 T-Type Calcium Channel Induced by Varicella-Zoster Virus Participates in the Maintenance of Herpetic Neuralgia

**DOI:** 10.3389/fneur.2021.741054

**Published:** 2021-11-30

**Authors:** Rongzhen Li, Mingxi Ou, Shaomin Yang, Jiabin Huang, Jiamin Chen, Donglin Xiong, Lizu Xiao, Songbin Wu

**Affiliations:** ^1^Department of Pain Medicine and Shenzhen Municipal Key Laboratory for Pain Medicine, Shenzhen Nanshan People's Hospital and the 6th Affiliated Hospital of Shenzhen University Health Science Center, Shenzhen, China; ^2^Department of Chemistry, University of Science and Technology of China, Hefei, China; ^3^Vanke Bilingual School, Shenzhen, China

**Keywords:** Herpetic neuralgia, T-type calcium channel, Cav3.2, VZV, dorsal root ganglion (DRG), spinal dorsal horn (SDH)

## Abstract

Pain, as the most prevalent neurological complication of herpes zoster (HZ), may occur before or during the rash onset or even after the rash has recovered. Particularly, postherpetic neuralgia (PHN) is a refractory chronic condition, usually defined as pain persisting for 3 months or longer from the onset of HZ. Pain evoked by HZ impairs the normal physical and emotional functions of the patients, severely reducing their quality of life. However, how zoster-associated pain occurs and develops into PHN are elusive, making PHN difficult to predict. Uncovering the pathogenesis of zoster-associated pain (or HN) helps us to better understand the onset of PHN and supports developing more effective treatments. In this study, we successfully constructed a model for zoster-associated pain through varicella-zoster virus (VZV) infections of mouse footpads and pain behavior assessments. Next, we used the Kyoto Encyclopedia of Genes and Genomes (KEGG) and the Gene Ontology (GO) to analyze PHN rodent dorsal root ganglion (DRG) gene microarray data and found that calcium signal disorder might be involved in the onset of PHN. By using reverse transcription real-time fluorescent quantitative PCR (RT-qPCR) and Western blotting, we confirmed that VZV infection could significantly upregulate the expression of T-type calcium channel Cav3.2 in DRG and spinal dorsal horn (SDH). Intrathecal administration of Cav3.2 blocker (2R/S)-6-prenylnaringenin (6-PNG) relieved mechanical and thermal hyperalgesia induced by VZV. Taken together, our data indicated that VZV might participate in the occurrence and development of HN by upregulating the expression of Cav3.2 in DRG and SDH. These findings will help to reveal the underlying mechanisms on long-lasting pain and PHN formation, providing a new insight that Cav3.2 can be the promising drug target for remitting PHN.

## Introduction

Herpes zoster (HZ) episodes, after the reactivation of latent varicella-zoster virus (VZV) in the sensory ganglia, are accompanied by rash and severe acute pain (1). About a quarter of the population suffers from HZ and associated complications throughout their lifetimes, with incidence climbing with age and/or reduced cellular immune response (2, 3). Most patients with HZ completely recover after several weeks and their acute pain can be relieved by early antiviral therapies. However, some still experience chronic pain and 9–14% of patients develop postherpetic neuralgia (PHN) suffering from refractory pain sensation for months or even years after the skin lesion heals (4). This may lead to disparate secondary consequences that affect the quality of life, causing depression, withdrawal from society, and even suicide (5, 6). Some evidence has proved that virus replication is not essential to induce ongoing pain, which explains the chronic pain after the rash is resolved. Therefore, it is urgent to elucidate the pathogenesis of zoster-associated pain and the development of PHN. Despite several identifications of risk factors of PHN (7) and promising novel drugs (8, 9), the treatment for HN, especially PHN, remains temporary and unsatisfactory. Better understanding of HN will help to identify meaningful targets and develop new treatments.

Voltage-gated Ca^2+^ channels (VGCCs) are formed by four distinct subunits (α1, α2δ, β, and γ). The α1-subunit forms a functional Ca^2+^ channel and possesses the main characteristics of the channel such as ion-conducting pore, ion selectivity, and voltage sensitivity (10, 11). The α2δ-subunit affects the biophysical properties and expression of the channel (12, 13). The β-subunit mediates different shifts in the kinetics and voltage dependence of gating by binding to the α1-subunit (12) and the γ-subunit is involved in modulating the biophysical properties of the channel (13). According to the main biophysical and pharmacological properties, VGCCs are classified into L-, N-, P/Q-, R-, and T-type subtypes (14). Among them, T-type calcium channels are activated by weak depolarization and consist of three subtypes based on the pore-forming α1-subunit: Cav3.1 (α1G), Cav3.2 (α1H), and Cav3.3 (α1I) (15, 16). Low-voltage-activated T-type calcium channels contribute to the exocytosis evoked by low voltage, serving as key regulators of neuronal excitability in the peripheral and central nervous systems (17, 18). They have been reported to play a crucial role in painful pathological conditions such as complete Freund's adjuvant- or formalin-induced inflammatory pain (19, 20), chronic constriction injury (CCI)- or spinal nerve ligation (SNL)-induced neuropathic pain (21, 22), and painful diabetic neuropathy (PDN) (23). However, there is little evidence to indicate the role of the T-type calcium channels in the zoster-associated neuralgia and development of PHN currently.

Hence, we downloaded the gene expression profile data set of the PHN model from the Gene Expression Omnibus (GEO) database and obtained preliminary evidence that calcium signals were involved in PHN by the Gene Ontology (GO) enrichment and the Kyoto Encyclopedia of Genes and Genomes (KEGG) analysis of the differentially expressed genes (DEGs) between PHN rats and the normal control group. Next, we developed HN murine models infected with VZV to investigate the changes of T-type calcium channels in dorsal root ganglion and spinal dorsal horn after the onset of HN to confirm the important role of Cav3.2 channels in VZV-induced HN.

## Materials and Methods

### Culture of Cells and Viruses

Human neuroblastoma cell line (SH-SY5Y) and Human retinal pigment epithelial cell line (ARPE-19) cells were purchased from American tissue culture colection (ATCC) and cultured in Dulbecco's modified Eagle medium (DMEM) containing 10% fetal bovine serum (Gibco, USA), 100 μg/ml streptomycin, 100 U/ml penicillin (Life Technologies, USA), and placed in 37°C, 5% carbon dioxide (CO_2_) atmosphere. The VZV-rOka strain was provided by Tong Cheng (Development in Infectious Diseases, Xiamen University, Xiamen, China). The preparation method of VZV virus particles is as follows: ARPE-19 monolayer cells were infected by VZV and the supernatant and cell particles were collected when 80–90% of the cells undergo cytopathic (cell rounding, swelling, and lysis) (24). The cells were disrupted by ultrasound (20 kHz, 45% amplitude, 15 s) and centrifuged at low speed (1,000 g, 5 min, 4°C) to remove cell debris. Collect the cell lysate supernatant and concentrate the virus particles by high-speed centrifugation (80,000 g, 3 h, 4°C). The VZV virus precipitate obtained by centrifugation was resuspended in an appropriate amount of DMEM and the virus titer was determined by the plaque test. All the virus-related experiments were carried out in the biosafety laboratory. The design and implementation of these experiments have obtained ethical and safety approval.

### Animal

All the animal studies were conducted according to procedures approved by the Shenzhen Nanshan People's Hospital and the Sixth Affiliated Hospital Animal Care and Use Committee. A total of 8-week-old male C57BL/6J mice with an average body weight of 25 ± 1 g (purchased from Guangdong Medical Laboratory Animal Center: No.119, Poyang Road, Huangqi, Nanhai, Foshan, Guangdong, China) were selected. Experimental animals were housed under individually ventilated colony cages and maintained in a normal 12/12 h light/dark cycle, with free access to food and water. All the animals were operated on and cared for in accordance with the guidelines of the International Association for the Study of Pain research on the use of animals in pain research.

### Preparation and Injection of Viral Inoculum

According to previous reports, the HN model was established by subcutaneous inoculation of VZV into the right hindfoot pad of mice and the injurious behaviors of mechanical hyperalgesia (MA) and thermal hyperalgesia (TH) were evaluated (25). VZV was propagated in human retinal epithelial cells (ARPE-19) and harvested when the cells show about 80% cytopathic effect (CPE) under the microscope. The virus-infected cells were gently scraped from the surface of the culture flask and the cell suspension was centrifuged at 1,000 rpm and 4°C for 10 min. The cell precipitates obtained from every 75 cm^2^ of culture bottle were suspended in 100 μl sterile phosphate buffer solution (Gibco). The 20 μl virus-infected cell suspension (about 2 × 10^5^ cells) was subcutaneously injected (SC) into the right (ipsilateral) hindfoot pad of mice. The control group was inoculated with ARPE-19 cell suspension with the same volume and cell density.

### Nociceptive Behavior Test

As previously described, von Frey filaments were used to assess the mechanical injury threshold in mice (26). About 30 min prior to the test, the mice were placed in acrylic cages (12 cm × 10 cm × 17 cm) with a wire mesh floor at the bottom. In these experiments, a series of von Frey filaments with logarithmically increased stiffness (0.008–2 log force, mg) were applied vertically to the middle of the right hind paw pad in a gradually increasing pressure manner. These tests elicited reactions including flexion, leg raising, or foot licking, followed by significant withdrawal after claw withdrawal. Each von Frey filament was applied for 3–4 s to induce terminal reflex. The smallest filaments that can cause reactions were considered to be the mechanical injury threshold.

The mice were placed in the hot plate test room for 30 min to adapt to the environment in advance. The mice were placed in the acrylic cage on the heating plate (the constant temperature was 53°C) and the latency of the first nociceptive reaction (licking the hind paw, shaking the hind paw, jumping, and spinning) was measured (27). In order to avoid tissue damage caused by high temperature, the cutoff time was 20 s.

### Intrathecal Injection of Drugs

Male C57BL/J mice weighing 25 ± 1 g were selected and intrathecal drug injection was performed 7 days after VZV inoculation. The intrathecal injection method of Cav3.2 blocker 6-prenylnaringenin [(2R/S)-6-PNG] [HY-115681, MedChemExpress, Princeton, New Jersey, USA] was as follows: Hair removal on the back of mice was accomplished by a razor and the bared skin was cleaned by a cotton ball containing 75% ethanol. The pelvic girdle of mice was held safely in one hand. Then, the intrathecal injection was conducted by putting a 25-μl microsyringe with a 30-Ga needle perpendicular to the vertebrae and inserting the needle into the tissue between the dorsal sides of L5 and L6. When the needle entered the subarachnoid space, the mice tail suddenly trembled or swung laterally, indicating a successful intrathecal injection. Ten microlitre of normal saline or normal saline containing different doses (2R/S)-6-PNG (5/25/50 μg) was injected into the mouse sheath and the syringe was held for a few seconds and carefully removed to avoid drug outflow (28).

### Total RNA Extraction

Dorsal root ganglia (DRG) and spinal dorsal horn (SDH) tissues were dissected from the mice: after euthanasia, the skin and muscle of the back were cut down to expose the spine and the spinous process and transverse process of the spine were gently stripped with bone cutting forceps. After exposing the SDH, the SDH was lifted with forceps to identify the enlarged DRG at the posterior root of the spinal nerve and its spinal canal. Lumbar L4-L5 DRG and ipsilateral L4-L5 SDH tissue were collected and put into a 1.5-ml centrifuge tube. Finally, the steel ball was put into the collected tissue and ground in the grinder at low temperature and then dissolved in the TRIzol reagent (Invitrogen, USA). For the cell experiment, ARPE-19 and SH-SY5Y were seeded on six-well cell plates (6 × 10^5^ cells/ml). On the next day, use 1 × 10^4^ Plaque forming unit (PFU)/ml acellular VZV-infected cells. After VZV infection for 24, 48, and 72 h, the cells were harvested and dissolved in the TRIzol reagent. RNA dissolved in the TRIzol reagent was extracted according to the manual of the manufacturer.

### RT-qPCR for Messenger RNA Quantification

The total RNA of each sample was treated with DNase I (Thermo Fisher Scientific, USA) to remove the genome. The synthesis of complementary DNA (cDNA) from 500 ng of total RNA was reverse transcribed by using the RevertAid™ Master Mix Kit (Thermo Fisher Scientific, USA). The primer sequence was referenced from the online website of PrimerBank (https://pga.mgh.harvard.edu/primerbank/index.html) and the specificity was verified by the primer basic local alignment search tool (BLAST) tool of the National Center for Biotechnology Information (NCBI) (Table 1). Gene expression was detected by RT-qPCR by using 2 × RealStar Green Fast Mixture [with Carboxy-X-rhodamine (ROX)] (GenStar, China) following the manual of the manufacturer. Data were collected and analyzed by using ABI QuantStudio™ version 5 software (Applied Biosystems, USA). The results were normalized to Glyceraldehyde-3-phosphate dehydrogenase *(GAPDH)* (human)*/Gapdh* (mouse) and relative expression was shown as 2^−Δ*ΔCt*^.

**Table 1:** Sequences of the primer for quantitative real-time PCR (RT-PCR).

**Gene name**	**Forward primer (5–3^**′**^)**	**Reverse primer (5–3^**′**^)**
*ORF61* (VZV)	ACATCCCTGCGTTGTCTTT	TTGAGGTGGTTTCTGGTCTTA
*GAPDH* (Human)	CTGGGCTACACTGAGCACC	AAGTGGTCGTTGAGGGCAATG
*HCav3.1* (Human)	TTGCCTACGGCTTCTTATTCCA	GTTCCAGAATCACGGTGAAGAC
*MCav3.1* (Mouse)	TCAGCATCGTGGAATGGAAAC	GTTCAGAGTGTTGTTGTCATCCT
*HCav3.2* (Human)	ATGCTGGTAATCATGCTCAACTG	AAAAGGCGAAAATGAAGGCGT
*MCav3.2* (Mouse)	ATGCTTGGGAACGTGCTTCTT	GTCTGGTAGTATGGCCGCAA
*HCav3.3* (Human)	ACTGGAACCGTTACTACAATGTG	GGAGTGAGCATCCATCACGTA
*MCav3.3* (Mouse)	CCATGTGACGATATGGAGTGC	CAGGTTGATGTTCTGTAGGTCC
*Gapdh* (Mouse)	TGGCCTTCCGTGTTCCTAC	GAGTTGCTGTTGAAGTCGCA

### Western Blot Analysis

Steel balls were added to the DRG and SDH collected in the centrifuge tube, adding precooled tissue lysate (Beyotime Biotechnology, China). The mixture was grounded in a precooled tissue grinder, centrifuged at 4°C and 12,000 rpm for 10 min, and then the supernatant was collected. ARPE-19 and SH-SY5Y cells were treated with 1 × 10^4^ PFU/ml cell-free VZV for 24, 48, and 72 h, respectively. The monolayer cells were scraped and centrifuged at 4°C and 1,000 rpm for 5 min to obtain cell precipitates. An appropriate amount of precooled western cell lysate (Beyotime Biotechnology, China) was added to the cell pellet. After the cell particles were disintegrated by the lysate, the mixture was centrifuged at 4°C and 12,000 rpm for 10 min, and the supernatant was collected. The total protein in tissue or cell lysates was quantitatively detected by Bicinchoninic acid (BCA) protein concentration assay kit (Beyotime Biotechnology, China) and the total protein was separated by 10% 12 sodium dodecyl sulfate-polyacrylamide gel electrophoresis (SDS-PAGE). The protein was then transferred to a polyvinylidene fluoride (PVDF) membrane (Millipore, USA), which would later be blocked with 5% nonfat dry milk (Bio-Rad Laboratories, USA) dissolved in Tris buffered saline with Tween (TBS-T) for 1 h at room temperature. Different antibodies were used to detect proteins carried on different membranes and incubated at 4°C overnight: anti-VZV glycoprotein E (gE) antibody (ab272686) (Abcam, USA; 1:2000), anti-CACNA1H antibody (ab135974) (Abcam, USA; 1:2000), anti-α-tubulin (#3873) (Cell Signaling Technology, USA; 1:2000), and anti-β-actin (#4970) (Cell Signaling Technology, USA; 1:2000). On the next day, after washing three times with TBS-T, the membrane was afterward incubated with an antirabbit or antimouse horseradish peroxidase (HRP)-conjugated antibody (CST, USA, 1:5000). Efficient chemiluminescence (ECL) Substrate Kit (Millipore, USA) was used to observe the expression of the target protein. At least three samples were analyzed by densitometry by using the (ImageJ software, USA) and corrected for loading by normalization to corresponding bands for Gapdh.

### Bioinformatics Analysis of *PHN* Gene Expression Profile

We downloaded the gene expression profile data set of PHN model [GSE64345 (GPL1355 platform)] from the GEO database (https://www.ncbi.nlm.nih.gov/geo/), an open-source platform for retrieving and storing gene sequencing data (29). The DEGs of DRG between PHN rats and the normal control group were screened and identified by the GEO2R online tool (https://www.ncbi.nlm.nih.gov/geo/geo2r/). The results of *p* < 0.05 and fold change (FC) > 1 or FC <1 were statistically significant and were defined as DEGs. Functional classification of DEGs between groups was performed by using the DAVID (USA) version 6.8 (https://david.ncifcrf.gov/) (30) and the KOBAS (China) version 3.0 (http://kobas.cbi.pku.edu.cn/kobas3) (31) online database.

### Statistical Analysis

All the quantitative data were presented as mean ± SEM from three or more independent experiments. Biochemical indicators and behavioral data were analyzed by using the one-way ANOVA or the two-way ANOVA followed by the *post-hoc* Bonferroni test and the statistical significance was analyzed by the GraphPad Prism version 9 (GraphPad Software, La Jolla, California, USA). When the *p*-value of the statistical variable is < 0.05, the data were considered to be statistically significant.

## Results

### Behavioral Characterization of the HN Mice

**Figure 1:**
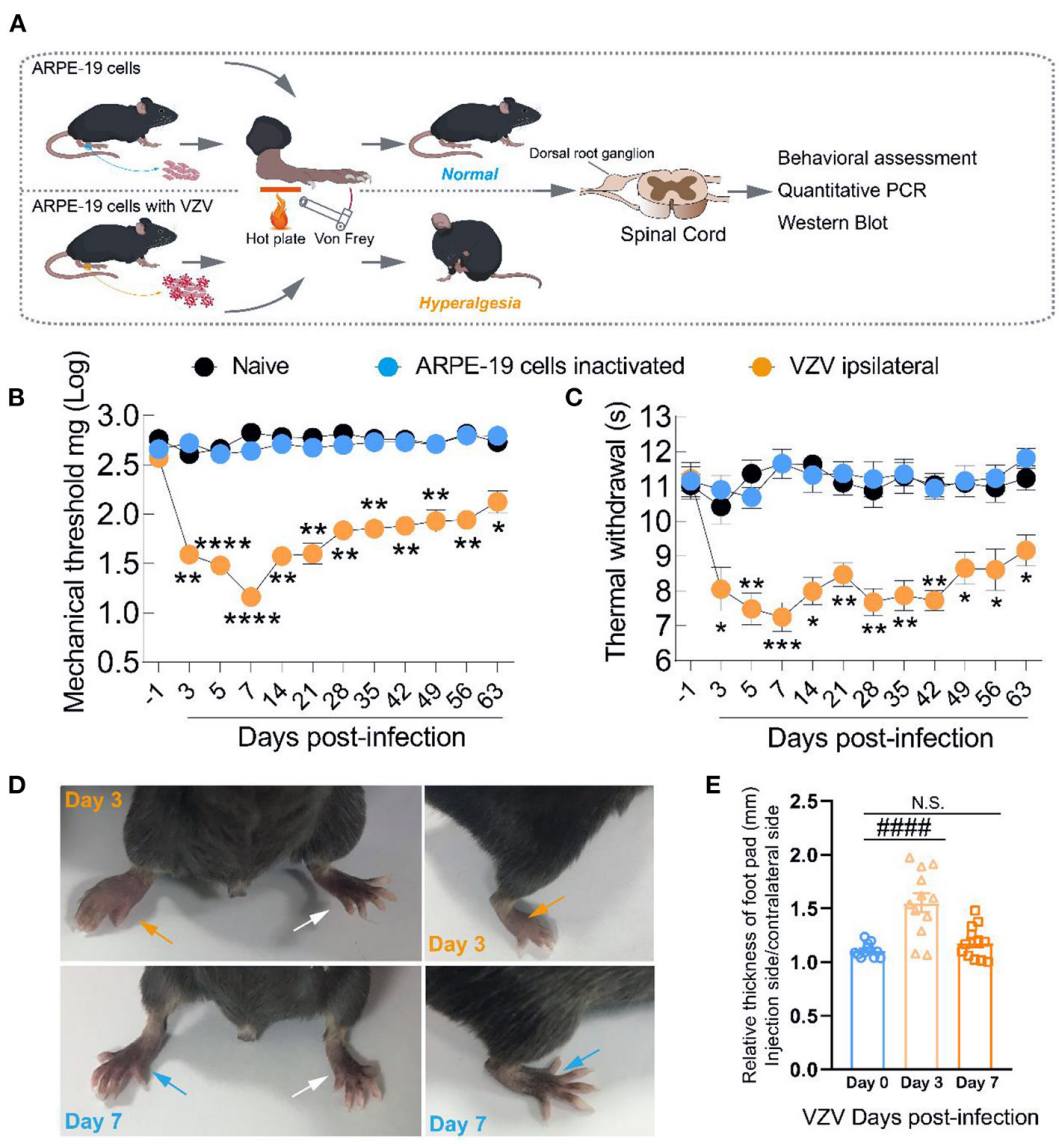
Intraplantar inoculation of varicella-zoster virus (VZV) evoked mechanical and heat hyperalgesia in mice. **(A)** Schematic of the experimental process. ARPE-19 cells or equivalent ARPE-19 cells with VZV were resuspended with phosphate-buffered saline (PBS) and inoculated into the glabrous region of the right rear footpad of male mice. The mechanical and heat allodynia after cell inoculation were tested with von Frey filaments and a hot plate. **(B,C)** Time-dependent changes in mechanical allodynia and thermal hyperalgesia of C57BL/6 mice over 63 days after normal control (naïve), inactivated cells, and VZV inoculation, *n* = 6. **(D)** Representative microphotographs of the inoculated and contralateral hind paws on days 3 and 7 after VZV inoculation. **(E)** Comparison of paw thickness on day 3 and day 7, *n* = 12. Yellow arrows: ipsilateral hind paws on day 3 after VZV inoculation; blue arrows: ipsilateral hind paws on day 7 after VZV inoculation; white arrows: the contralateral hind paws; *p < 0.05, **p < 0.01, ***p < 0.001, ****p < 0.0001, compared with APPE-19 cells inactivated; ^*####*^*p* < 0.0001, compared with before VZV inoculation.

We inoculated ARPE-19 cells with VZV into the right rear footpad of male mice to create an HN model. The schematic detailed experimental process was shown in Figure 1A. The mechanical and heat thresholds were tested by using von Frey filaments and a hot plate. Mice inoculated with ARPE-19 cells with VZV showed mechanical (Figure 1B) and thermal allodynia (Figure 1C), which peaked at 5 or 7 days after VZV inoculation. The allodynia continued until day 63 (Figures 1B,C), mimicking the chronic condition of long-lasting neuralgia that may further form PHN. VZV inoculation caused redness and swelling on the ipsilateral side, which disappeared within 7 days after virus inoculation (Figures 1D,E). This finding confirmed that it was VZV infection, rather than the inflammatory response, induced allodynia in mice.

To further confirm that the mouse inoculum is VZV, we infected the mouse inoculum *in vitro* with ARPE-19 and SH-SY5Y cells, which are generally considered to be induced the lytic infection by VZV. Two cell lines showed obvious cytopathic effects at 24, 48, and 72 h after infection, while the morphology of uninfected cells virus free control group (MOCK) remains normal (Supplementary Figure S1A). Moreover, the expression of the *ORF61* gene (encoding the virus early phosphorylation protein) and VZV membrane gE were detected by qRT-PCR and Western blot, respectively (Supplementary Figures S1B,C). The above results suggested that the HN mice model was successfully constructed.

### Varicella-Zoster Virus Infection Increased the Expression of Cav3.2 in Mice DRG and SDH

The GO and the KEGG results showed that biological events such as “calcium signaling pathway,” “calcium-mediated signaling,” and “calcium ion binding” confirmed that the calcium signal disorder was related to the occurrence of PHN (Supplementary Figures S2, S3). As an important member of the calcium channel family, low-voltage activated calcium channels, namely T-type calcium channels, are generally considered to be the key regulators of neuronal excitability in the peripheral and central nervous systems (15, 17). The quantitative results of gene microarray data showed that the quantification level of *Cav3.2*, instead of *Cav3.1* or *Cav3.3* genes, was higher in the DRG of the PHN group than that of the sham group (Supplementary Figure S4).

**Figure 2:**
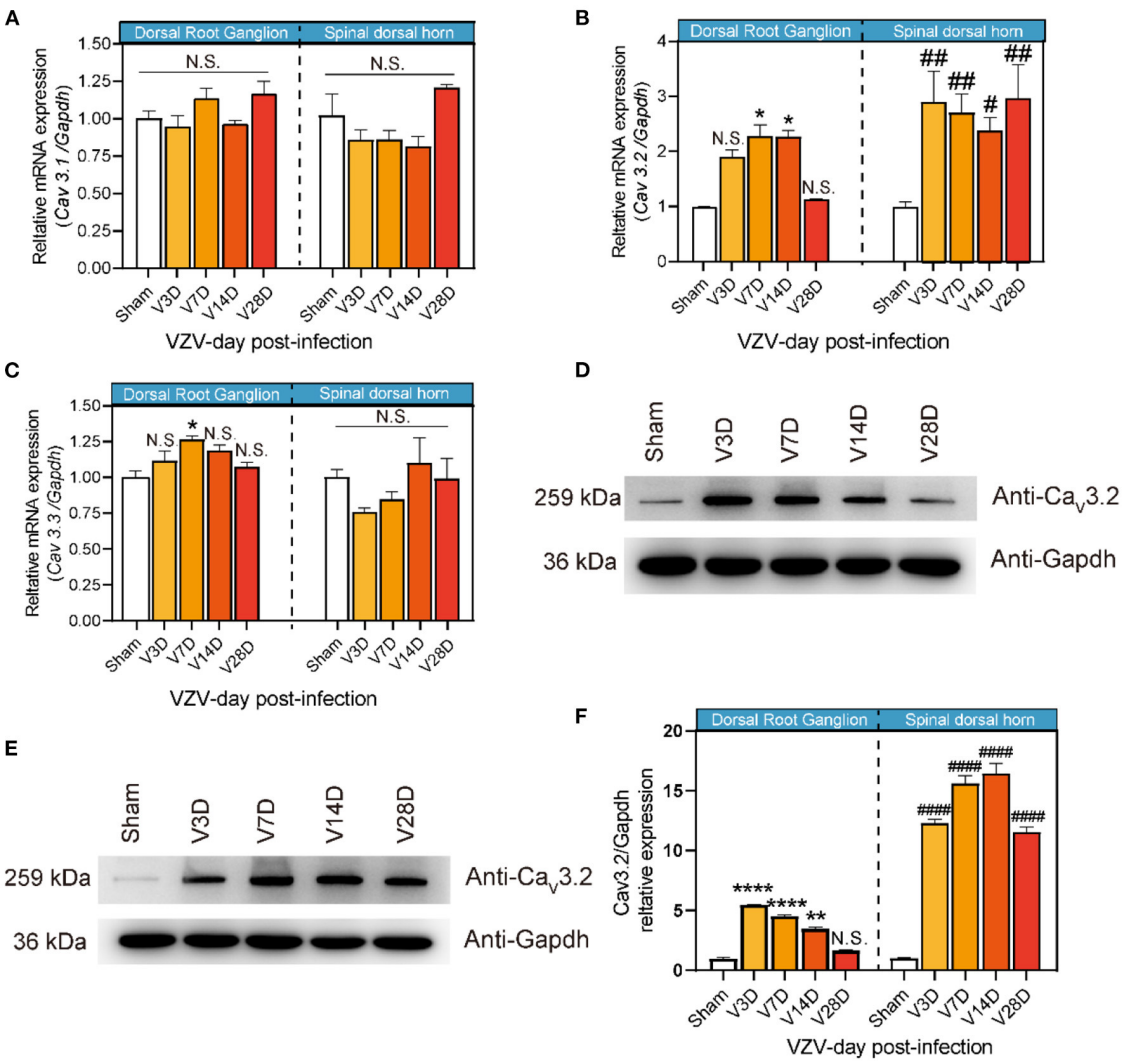
Expression of Cav3.1, Cav3.2, and Cav3.3 in dorsal root ganglion and spinal dorsal horn in sham- and VZV-infected mice. RT-qPCR results of *Cav3.1* **(A)**, *Cav3.2* **(B)**, and *Cav3.3* **(C)** messenger RNA (mRNA) expression in dorsal root ganglion and spinal dorsal horn relative to *Gapdh* in sham- and VZV-infected mice. Western blot results of Cav3.2 and Gapdh protein expression in dorsal root ganglion **(D)** and spinal dorsal horn **(E)** in sham- and VZV-infected mice. **(F)** Relative quantitative results of Cav3.2/Gapdh protein expression. *p < 0.05, **p < 0.01, ****p < 0.0001 compared with sham mice in dorsal root ganglion. ^#^*p* < 0.05, ^*##*^*p* < 0.01, ^*####*^*p* < 0.0001 compared with sham mice in spinal dorsal horn; NS, not significant. V3D, V7D, V14D, and V28D represent days 3, 7, 14, and 28 after VZV inoculation, respectively, *n* = 6.

Therefore, we tried to further clarify the dynamic changes of T-type calcium channels in the DRG and SDH from the HN mice model induced by VZV. As shown in Figure 2B, *Cav3.2* mRNA in the SDH of VZV mice increased significantly compared to the sham mice on day 3 after VZV infection and the upregulation persisted until day 28 and the mRNA expression on day 28 did not return to its original level, implying that this trend may continue. *Cav3.2* mRNA in DRG of VZV mice also increased significantly compared to the sham mice on days 7 and 14 after VZV infection. However, no significant difference was found in mRNA levels of *Cav3.1* and *Cav3.3* between the two groups, except for the slight increase of *Cav3.3* in DRG on day 7 after VZV infection (Figures 2A,C). Consistently, Western blot analysis showed that the protein level of Cav3.2 increased after VZV infection in mice DRG and SDH (Figures 2D-F).

We also infected ARPE-19 and SH-SY5Y cells with VZV *in vitro* and conducted Western blot analysis, showing that the protein level of *Cav3.2* in ARPE-19 and SH-SY5Y cells increased after VZV infection (Supplementary Figure S5D). The expression of *Cav3.2* mRNA in SH-SY5Y cells also increased after VZV infection, but this trend was not detected in ARPE-19 cells (Supplementary Figure S5B), which was probably due to the low expression of *Cav3.2* mRNA in ARPE-19. Interestingly, *Cav3.1* mRNA was not detected in ARPE-19 and SH-SY5Y cells after VZV infection, while the expression of *Cav3.3* increased in ARPE-19 cells (Supplementary Figures S5A,C).

### Blocking the Cav3.2 Channel Reduces Mechanical Allodynia and Thermal Hyperalgesia in HN Mice

Given that the calcium signal disorder in the DRG and Cav3.2 was significantly upregulated in both the DRG and SDH of PHN mice, we speculated that Cav3.2 might be a key factor involved in mechanical and thermal hyperalgesia during VZV-induced neuralgia. (2R/S)-6-PNG, a Cav3.2 blocker, can potently block Cav3.2, but exhibit a minor effect on high-voltage activated Ca^2+^ channels and voltage-gated Na^+^ channels (32). VZV was inoculated into the right hind paw of the mouse to induce the HN mouse model and then (2R/S)-6-PNG was intrathecally injected on day 7 after VZV inoculation and the analgesic effect was evaluated.

**Figure 3:**
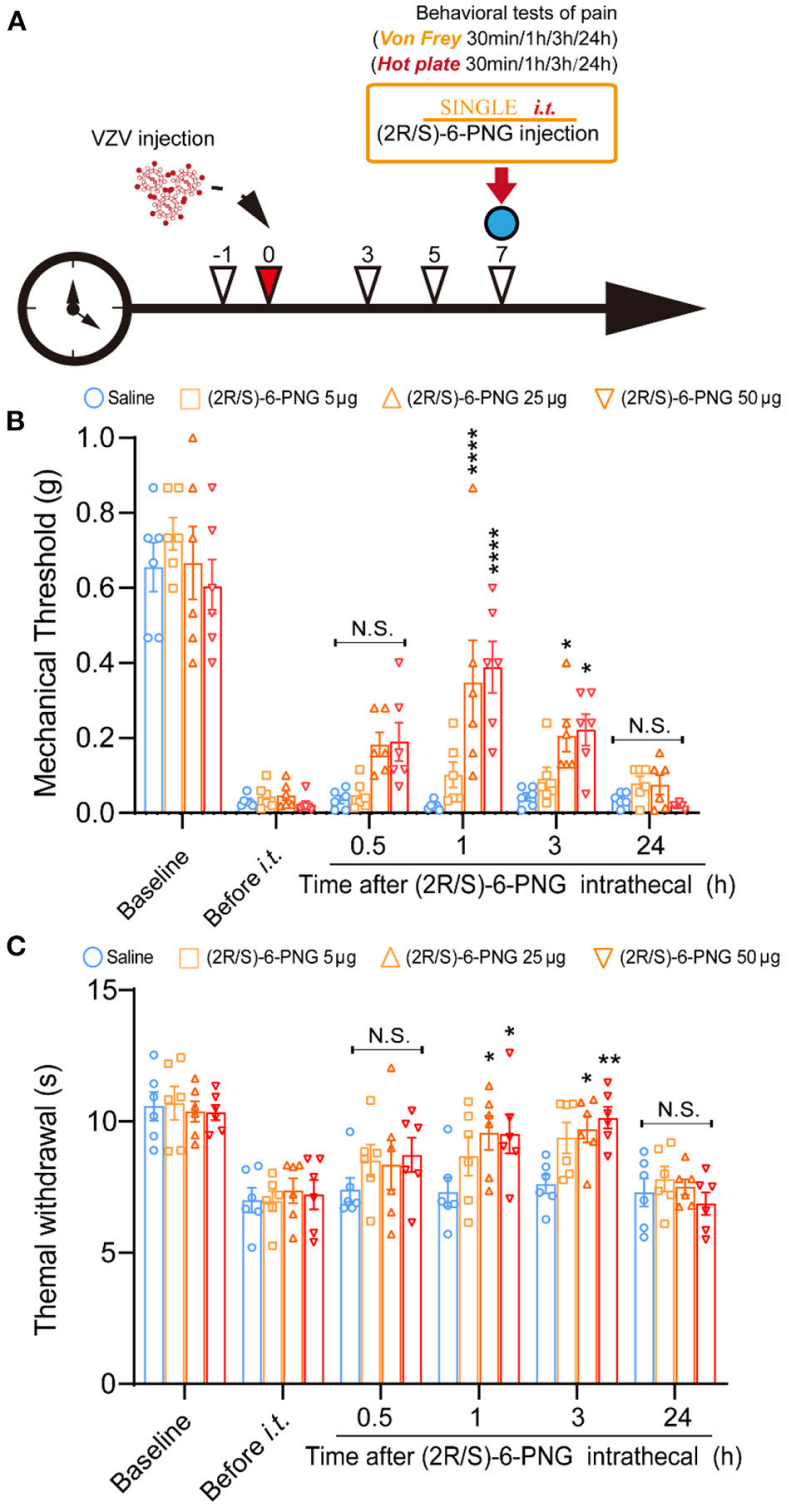
Intrathecal injection of (2R/S)-6-PNG (Cav3.2 inhibitor) alleviated mechanical and heat hyperalgesia in postherpetic neuralgia (PHN) mice. **(A)** Schematic operation process. VZV was inoculated into the footpad of male mice on day 0, then intrathecal injection of (2R/S)-6-PNG was performed on day 7 after VZV inoculation. Mechanical and heat hyperalgesia before and after VZV inoculation, 0.5, 1, 3, and 24 h after (2R/S)-6-PNG injection were tested with von Frey filaments and a hot plate. **(B)** Alleviated mechanical hyperalgesia occurred in 1 and 3 h after (2R/S)-6-PNG (25 and 50 μg) injection. **(C)** Alleviated heat hyperalgesia occurred in 1 and 3 h after (2R/S)-6-PNG (25 and 50 μg) injection. *p < 0.05, **p < 0.01, ****p < 0.0001 compared with the saline group at the corresponding time; NS, not significant; *it*, intrathecal; *n* = 6.

Behavior tests were performed to assess mechanical and thermal sensitivity of HN mice at 30 min, 1, 3, and 24 h after intrathecal injection of (2R/S)-6-PNG (Figure 3). The schematic detailed operation process is shown in Figure 3A. Before (2R/S)-6-PNG injection, PHN mice exhibited a reduction in paw withdrawal threshold in von Frey test and hot plate test, while the increase in the threshold occurred at 1, and 3 after (2R/S)-6-PNG injection (Figures 3B,C). Collectively, these results indicated that blocking the Cav3.2 channel can alleviate neuropathic pain induced by VZV.

## Discussion

In clinics, there are many interventional ways to treat early HZ neuralgia such as medication, physical therapy, and surgical treatment (33). However, some patients with shingles develop PHN and their chronic pain cannot be completely cured. The fundamental reason is the complexity and uncertainty of the pathogenesis of PHN, which leads to the frustrating effect of clinical treatments for HN, especially PHN. In this study, we established a model of HN by inoculating with VZV under the footpads of mice and verified its reliability by the mechanical and thermal allodynia behavioral tests. At the same time, we measured the relative thickness of the inoculated side and contralateral footpads of the mice to rule out the allodynia caused by acute inflammation after virus inoculation, confirming that VZV infection was able to induce long-term neuropathic pain.

In order to obtain an insight into the most disturbing HN, PHN, we obtained PHN rodent DRG gene microarray data from the GEO database and used bioinformatics to explore possible pathogenesis. Compared with the control group, the results of the KEGG and the GO analysis showed that the biological functions of the DEGs in PHN rodent DRG are mainly involved in the calcium signal pathways. Calcium ion is well known as the indispensable second messenger in various physiological activities including the generation and transmission of pain-related signals between neurons (34). Although the exact molecular and cellular mechanisms of chronic pain may vary depending on the causes and types and have yet to be further elucidated, there is accumulating evidence on the role of intracellular Ca^2+^ in the development of persistent pain (35). In fact, there are a variety of voltage-gated and ligand-gated Ca^2+^ channels in the DRG, SDH, or glial (34, 36). These calcium channels and receptors have been reported to be involved in pain and neuroplasticity associated with chronic pain states (35). In particular, our previous studies have shown that VZV infection could cause cellular calcium signal disorders manifested by significant changes in the expression of calcium signal-related genes and abnormalities in the distribution and content of calcium ions in cells (37). All the evidence implied that VZV-mediated abnormal expression and/or dysfunction of cellular calcium channels and receptors might be an important factor in the onset and maintenance of PHN.

T-type Ca^2+^ channel has been reported as a key regulator of neuronal excitability in the peripheral and central nervous system, contributing to the transmission of neural signals induced by low voltage (17, 38). It was first reported in peripheral sensory neurons of the DRG and subsequently confirmed to be involved in excitatory transmission in the thalamus and corticothalamic neurons (39, 40). Consistently, our data showed that T-type calcium channels were expressed in mouse DRG and SDH. The time-dependent Cav3.2 expression pattern induced by VZV was surprisingly synchronized with the changes in mice pain sensitivity after VZV inoculation, showing an increasing trend first and then followed by a decrease. Intrathecal injection of Cav3.2 blocker (2R/S)-6-PNG (25 or 50 μg) significantly upregulated the reduction of mechanical and thermal hyperalgesia induced by VZV within 1–3 h. Therefore, we speculated that VZV might mediate HN by upregulating the expression of Cav3.2 and how to extend this conclusion to PHN development remains a matter of discussion.

In fact, the role of Cav3.2 in neuropathic pain has been extensively studied by behavior tests, electrophysiology, and molecular biology methods. Accompanied by enhanced T-type Ca^2+^ currents, Cav3.2 expression significantly increases in the SDH and DRG neurons under the pathological conditions related to chronic pain such as peripheral nerve injury or inflammation, diabetic neuropathy, and paclitaxel-induced peripheral neuropathy (41–44). In addition, blocking Cav3.2 channels by T-type calcium channel blockers, intrathecal injection of antisense oligonucleotides, or conditional gene deletion produced strong analgesic effects in rodent pain models (21, 45, 46). It remains elusive how the T-type Ca^2+^ channels are involved in the signal transmission of pain. Given that Cav3.2 is widely expressed in the tissues including peripheral pain endings, skin afferent nerve axons, Aδ fibers, and nociceptive fibers in the spinal cord (38, 46, 47), we speculated that mediating the generation of instantaneous explosive potentials and altering the generation and propagation of action potentials could be the underlying mechanism of peripheral or central sensitization associated with the development and maintenance of chronic pain (48).

## Conclusion

In this study, we established an HN model and verified that VZV inoculation of mouse footpads induced long-term mechanical and thermal pain sensitivity similar to PHN. Gene microarray data implied the involvement of calcium signal disorder in VZV-induced PHN, which was supported by our qRT-PCR and Western blotting results, confirming that VZV infection could significantly upregulate the expression level of Cav3.2 in DRG and SDH during the neuralgia. Intrathecal administration of Cav3.2 inhibitor (2R/S)-6-PNG alleviated the mechanical and thermal sensitivity of mice caused by VZV, further illustrating that Cav3.2 played a key role in zoster-associated pain and had the potential to be a novel drug target. Altogether, these findings will increase our understanding of the pathogenesis of zoster-associated neuralgia and PHN, promoting more effective treatments.

## Data Availability Statement

The original contributions presented in the study are included in the article/Supplementary Material, further inquiries can be directed to the corresponding author/s.

## Ethics Statement

The animal study was reviewed and approved by Shenzhen Nanshan People's Hospital and the 6th Affiliated Hospital of Shenzhen University Health Science Center Animal Care and Use Committee.

## Author Contributions

SW, DX, and LX contributed to the study supervision and coordination and the design of the experiments. SW and RL conducted and performed all the experiments. MO, SY, JC, and JH assisted various portions of the experiments and analysis of data. SW, RL, MO, and JC wrote the manuscript. All authors contributed to the article and approved the submitted version.

## Funding

This study has been supported by grants from the Nanshan District Health Bureau on 2019 health technology projects (No. 2019012), the Shenzhen Nanshan People's Hospital, and the Sixth Affiliated Hospital of Shenzhen University Health Science Center (No. NY2021008).

## Conflict of Interest

The authors declare that the research was conducted in the absence of any commercial or financial relationships that could be construed as a potential conflict of interest.

## Publisher's Note

All claims expressed in this article are solely those of the authors and do not necessarily represent those of their affiliated organizations, or those of the publisher, the editors and the reviewers. Any product that may be evaluated in this article, or claim that may be made by its manufacturer, is not guaranteed or endorsed by the publisher.

## References

[1] Schmader KE Dworkin RH. Natural history and treatment of herpes zoster. J Pain. (2008) 9:S3–9. 10.1016/j.jpain.2007.10.002 18166460

[2] Harpaz R Ortega-Sanchez IR Seward JF. Prevention of herpes zoster: recommendations of the advisory committee on immunization practices (ACIP). MMWR. (2008) 57:1–30. 18528318

[3] Johnson RW Rice AS. Clinical practice. Postherpetic neuralgia. N Engl J Med. (2014) 371:1526–33. 10.1056/NEJMcp1403062 25317872

[4] Watson CP Oaklander AL. Chapter 44 postherpetic neuralgia. Handb Clin Neurol. (2006) 81:661–77. 10.1016/S0072-9752(06)80048-5 18808866

[5] Drolet M Brisson M Schmader KE Levin MJ Johnson R Oxman MN. The impact of herpes zoster and postherpetic neuralgia on health-related quality of life: a prospective study. CMAJ. (2010) 182:1731–6. 10.1503/cmaj.091711 20921251 PMC2972323

[6] Schmader KE Johnson GR Saddier P Ciarleglio M Wang WW Zhang JH. Effect of a zoster vaccine on herpes zoster-related interference with functional status and health-related quality-of-life measures in older adults. J Am Geriatr Soc. (2010) 58:1634–41. 10.1111/j.1532-5415.2010.03021.x 20863322 PMC2946120

[7] Jung BF Johnson RW Griffin DR Dworkin RH. Risk factors for postherpetic neuralgia in patients with herpes zoster. Neurology. (2004) 62:1545–51. 10.1212/01.WNL.0000123261.00004.29 15136679

[8] Schutzer-Weissmann J Farquhar-Smith P. Post-herpetic neuralgia - a review of current management and future directions. Expert Opin Pharmacother. (2017) 18:1739–50. 10.1080/14656566.2017.1392508 29025327

[9] Xie F Li X Bao M Guo R Zhang C Wu A. Plerixafor may treat intractable post-herpetic neuralgia. Med Hypotheses. (2015) 85:491–3. 10.1016/j.mehy.2015.07.005 26175195

[10] Catterall WA. Structure and regulation of voltage-gated Ca2+ channels. Annu Rev Cell Dev Biol. (2000) 16:521–55. 10.1146/annurev.cellbio.16.1.521 11031246

[11] Hayashi K Wakino S Sugano N Ozawa Y Homma K Saruta T. Ca2+ channel subtypes and pharmacology in the kidney. Circ Res. (2007) 100:342–53. 10.1161/01.RES.0000256155.31133.49 17307972

[12] Klugbauer N Marais E Hofmann F. Calcium channel alpha2delta subunits: differential expression, function, drug binding. J Bioenerg Biomembr. (2003) 35:639–47. 10.1023/B:JOBB.0000008028.41056.58 15000524

[13] Arikkath J Campbell KP. Auxiliary subunits: essential components of the voltage-gated calcium channel complex. Curr Opin Neurobiol. (2003) 13:298–307. 10.1016/S0959-4388(03)00066-7 12850214

[14] Ertel EA Campbell KP Harpold MM Hofmann F Mori Y Perez-Reyes E. Nomenclature of voltage-gated calcium channels. Neuron. (2000) 25:533–5. 10.1016/S0896-6273(00)81057-0 10774722

[15] Catterall WA Perez-Reyes E Snutch TP Striessnig J. International Union of Pharmacology. XLVIINomenclature I, and structure-function relationships of voltage-gated calcium channels. Pharmacological reviews. (2005) 57:411–25. 10.1124/pr.57.4.5 16382099

[16] Iftinca MC Zamponi GW. Regulation of neuronal T-type calcium channels. Trends Pharmacol Sci. (2009) 30:32–40. 10.1016/j.tips.2008.10.004 19042038

[17] Weiss N Hameed S Fernandez-Fernandez JM Fablet K Karmazinova M Poillot C. A Ca(v)32/syntaxin-1A signaling complex controls T-type channel activity and low-threshold exocytosis. J Biol Chem. (2012) 287:2810–8. 10.1074/jbc.M111.290882 22130660 PMC3268438

[18] Chemin J Monteil A Perez-Reyes E Bourinet E Nargeot J Lory P. Specific contribution of human T-type calcium channel isotypes (alpha(1G), alpha(1H) and alpha(1I)) to neuronal excitability. J Physiol. (2002) 540:3–14. 10.1113/jphysiol.2001.013269 11927664 PMC2290209

[19] Bladen C McDaniel SW Gadotti VM Petrov RR Berger ND Diaz P. Characterization of novel cannabinoid based T-type calcium channel blockers with analgesic effects. ACS Chem Neurosci. (2015) 6:277–87. 10.1021/cn500206a 25314588 PMC4372069

[20] Gadotti VM You H Petrov RR Berger ND Diaz P Zamponi GW. Analgesic effect of a mixed T-type channel inhibitor/CB2 receptor agonist. Mol Pain. (2013) 9:32. 10.1186/1744-8069-9-32 23815854 PMC3703287

[21] Bourinet E Alloui A Monteil A Barrere C Couette B Poirot O. Silencing of the Cav32 T-type calcium channel gene in sensory neurons demonstrates its major role in nociception. EMBO J. (2005) 24:315–24. 10.1038/sj.emboj.7600515 15616581 PMC545807

[22] Lai CY Hsieh MC Ho YC Lee AS Wang HH Cheng JK. Growth Arrest and DNA-damage-inducible Protein 45β-mediated DNA Demethylation of Voltage-dependent T-type Calcium Channel 32 Subunit Enhances Neuropathic Allodynia after Nerve Injury in Rats. Anesthesiology. (2017) 126:1077–95. 10.1097/ALN.0000000000001610 28346321

[23] Orestes P Osuru HP McIntire WE Jacus MO Salajegheh R Jagodic MM. Reversal of neuropathic pain in diabetes by targeting glycosylation of Ca(V)32 T-type calcium channels. Diabetes. (2013) 62:3828–38. 10.2337/db13-0813 23835327 PMC3806612

[24] Jiang HF Wang W Jiang X Zeng WB Shen ZZ Song YG. ORF7 of varicella-zoster virus is required for viral cytoplasmic envelopment in differentiated neuronal cells. J Virol. (2017) 91:e00127–17. 10.1128/JVI.00127-17 28356523 PMC5446663

[25] Hasnie FS Breuer J Parker S Wallace V Blackbeard J Lever I. Further characterization of a rat model of varicella zoster virus-associated pain: relationship between mechanical hypersensitivity and anxiety-related behavior, and the influence of analgesic drugs. Neuroscience. (2007) 144:1495–508. 10.1016/j.neuroscience.2006.11.029 17197105 PMC2394505

[26] Silva JR Lopes AH Talbot J Cecilio NT Rossato MF Silva RL. Neuroimmune-Glia interactions in the sensory ganglia account for the development of acute herpetic neuralgia. J Neurosci. (2017) 37:6408–22. 10.1523/JNEUROSCI.2233-16.2017 28576938 PMC6596605

[27] Gross T Wack G Syhr KMJ Tolmachova T Seabra MC Geisslinger G. Rab27a contributes to the processing of inflammatory pain in mice. Cells. (2020) 9:1488. 10.3390/cells9061488 32570938 PMC7349490

[28] Mestre C Pelissier T Fialip J Wilcox G Eschalier A. A method to perform direct transcutaneous intrathecal injection in rats. J Pharmacol Toxicol Methods. (1994) 32:197–200. 10.1016/1056-8719(94)90087-6 7881133

[29] Guedon JM Yee MB Zhang M Harvey SA Goins WF Kinchington PR. Neuronal changes induced by varicella zoster virus in a rat model of postherpetic neuralgia. Virology. (2015) 482:167–80. 10.1016/j.virol.2015.03.046 25880108 PMC4461525

[30] Huang da W Sherman BT Lempicki RA. Systematic and integrative analysis of large gene lists using DAVID bioinformatics resources. Nat Protoc. (2009) 4:44–57. 10.1038/nprot.2008.211 19131956

[31] Xie C Mao X Huang J Ding Y Wu J Dong S. KOBAS 20: a web server for annotation and identification of enriched pathways and diseases. Nucleic Acids Res. (2011) 39:W316–22. 10.1093/nar/gkr483 21715386 PMC3125809

[32] Sekiguchi F Fujita T Deguchi T Yamaoka S Tomochika K Tsubota M. Blockade of T-type calcium channels by 6-prenylnaringenin, a hop component, alleviates neuropathic and visceral pain in mice. Neuropharmacology. (2018) 138:232–44. 10.1016/j.neuropharm.2018.06.020 29913186

[33] Makharita MY. Prevention of post-herpetic neuralgia from dream to reality: a ten-step model. Pain Physician. (2017) 20:E209–20. 10.36076/ppj.2017.E220 28158158

[34] Anderson M Zheng Q Dong X. Investigation of pain mechanisms by calcium imaging approaches. Neurosci Bull. (2018) 34:194–9. 10.1007/s12264-017-0139-9 28501905 PMC5799123

[35] Mei Y Barrett JE Hu H. Calcium release-activated calcium channels and pain. Cell Calcium. (2018) 74:180–5. 10.1016/j.ceca.2018.07.009 30096536 PMC6119525

[36] Simms BA Zamponi GW. Neuronal voltage-gated calcium channels: structure, function, and dysfunction. Neuron. (2014) 82:24–45. 10.1016/j.neuron.2014.03.016 24698266

[37] Wu S Yang S Ou M Chen J Huang J Xiong D. Transcriptome analysis reveals the role of cellular calcium disorder in varicella zoster virus-induced post-herpetic neuralgia. Front Mol Neurosci. (2021) 14:665931. 10.3389/fnmol.2021.665931 34079439 PMC8166323

[38] Jacus MO Uebele VN Renger JJ Todorovic SM. Presynaptic Cav32 channels regulate excitatory neurotransmission in nociceptive dorsal horn neurons. J Neurosci. (2012) 32:9374–82. 10.1523/JNEUROSCI.0068-12.2012 22764245 PMC3398424

[39] Petersen M Wagner G Pierau FK. Modulation of calcium-currents by capsaicin in a subpopulation of sensory neurones of guinea pig. Naunyn Schmiedebergs Arch Pharmacol. (1989) 339:184–91. 10.1007/BF00165142 2542804

[40] Huguenard JR Prince DA. Intrathalamic rhythmicity studied in vitro: nominal T-current modulation causes robust antioscillatory effects. J Neurosci. (1994) 14:5485–502. 10.1523/JNEUROSCI.14-09-05485.1994 8083749 PMC6577071

[41] Garcia-Caballero Gadotti VM Stemkowski P Weiss N Souza IA Hodgkinson V. The deubiquitinating enzyme USP5 modulates neuropathic and inflammatory pain by enhancing Cav3.2 channel activity. Neuron. (2014) 83:1144–58. 10.1016/j.neuron.2014.07.036 25189210

[42] Marger F Gelot A Alloui A Matricon J Ferrer JF Barrere C. T-type calcium channels contribute to colonic hypersensitivity in a rat model of irritable bowel syndrome. Proc Natl Acad Sci U S A. (2011) 108:11268–73. 10.1073/pnas.1100869108 21690417 PMC3131334

[43] Li Y Tatsui CE Rhines LD North RY Harrison DS Cassidy RM. Dorsal root ganglion neurons become hyperexcitable and increase expression of voltage-gated T-type calcium channels (Cav32) in paclitaxel-induced peripheral neuropathy. Pain. (2017) 158:417–29. 10.1097/j.pain.0000000000000774 27902567 PMC5303135

[44] Messinger RB Naik AK Jagodic MM Nelson MT Lee WY Choe WJ. *In vivo* silencing of the Ca(V)32 T-type calcium channels in sensory neurons alleviates hyperalgesia in rats with streptozocin-induced diabetic neuropathy. Pain. (2009) 145:184–95. 10.1016/j.pain.2009.06.012 19577366 PMC2735619

[45] Shiue SJ Wang CH Wang TY Chen YC Cheng JK. Chronic intrathecal infusion of T-type calcium channel blockers attenuates CaV32 upregulation in nerve-ligated rats. Acta Anaesthesiol Taiwan. (2016) 54:81–7. 10.1016/j.aat.2016.09.001 27765616

[46] Francois Schuetter N Laffray S Sanguesa J Pizzoccaro A Dubel S. The low-threshold calcium channel cav3.2 determines low-threshold mechanoreceptor function. Cell Rep. (2015) 10:370–82. 10.1016/j.celrep.2014.12.042 25600872

[47] Rose KE Lunardi N Boscolo A Dong X Erisir A Jevtovic-Todorovic V. Immunohistological demonstration of CaV32 T-type voltage-gated calcium channel expression in soma of dorsal root ganglion neurons and peripheral axons of rat and mouse. Neuroscience. (2013) 250:263–74. 10.1016/j.neuroscience.2013.07.005 23867767 PMC3796369

[48] Elies J Scragg JL Boyle JP Gamper N Peers C. Regulation of the T-type Ca(2+) channel Cav32 by hydrogen sulfide: emerging controversies concerning the role of H2 S in nociception. J Physiol. (2016) 594:4119–29. 10.1113/JP270963 26804000 PMC4967741

