# Supplementary material for: Change in Cav3.2 T-Type Calcium Channel Induced by Varicella-Zoster Virus Participates in the Maintenance of Herpetic Neuralgia
Source: Front Neurol. 2021 Nov 30;12:741054. doi: 10.3389/fneur.2021.741054 (PMC8671009; doi:10.3389/fneur.2021.741054)
Supplement: Supplementary file 1 [file Data_Sheet_1.DOCX]

**Supplementary materials**

Fig.S1


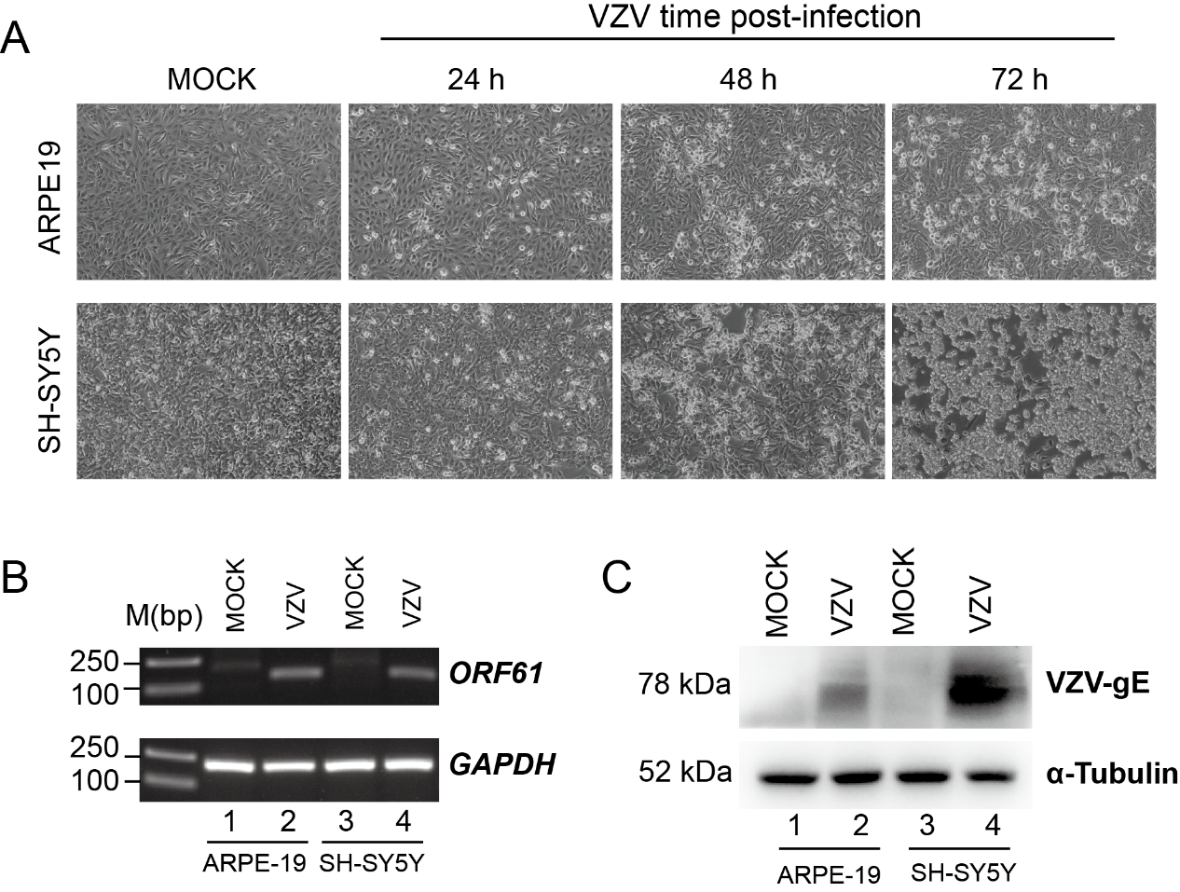


**Figure S1. The cytopathic changes of ARPE-19 and SH-SY5Y cells infected with VZV.** (A) ARPE-19 and SH-SY5Y cells induced obvious cytopathic effects at 24, 48 and 72 h after infected with VZV, while the morphology of uninfected cells (MOCK) remains normal. (B) Representative images of VZV transcript *ORF61* expression in ARPE-19 (left) and SH-SY5Y (right) cells on agarose gel. GAPDH was included as a loading control. (C) Western blot was used to evaluate the expression of VZV glycoprotein gE in ARPE-19 (left) and SH-SY5Y (right) cells.

Fig.S2


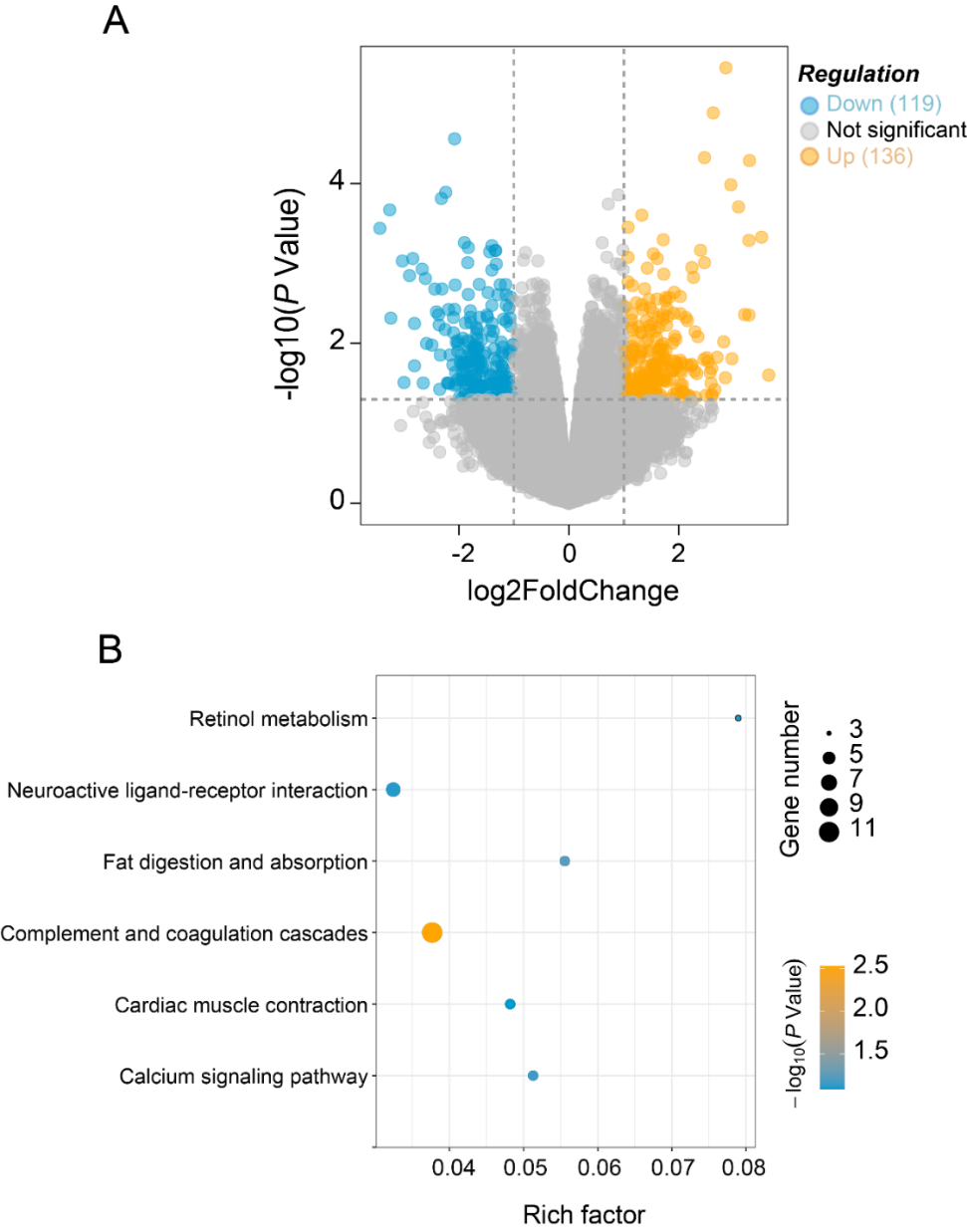


**Figure S2. The distribution and KEGG pathway enrichment analysis of DEGs.** (A) The volcanic map showed the number and distribution of DEGs in PHN rat model. The significantly upregulated genes are shown in orange and the significantly downregulated genes are shown in blue. (B) KEGG pathway enrichment analysis of DEGs in PHN rat model. The rich factor is plotted as the abscissa and KEGG terms are plotted as the ordinate.

Fig.S3


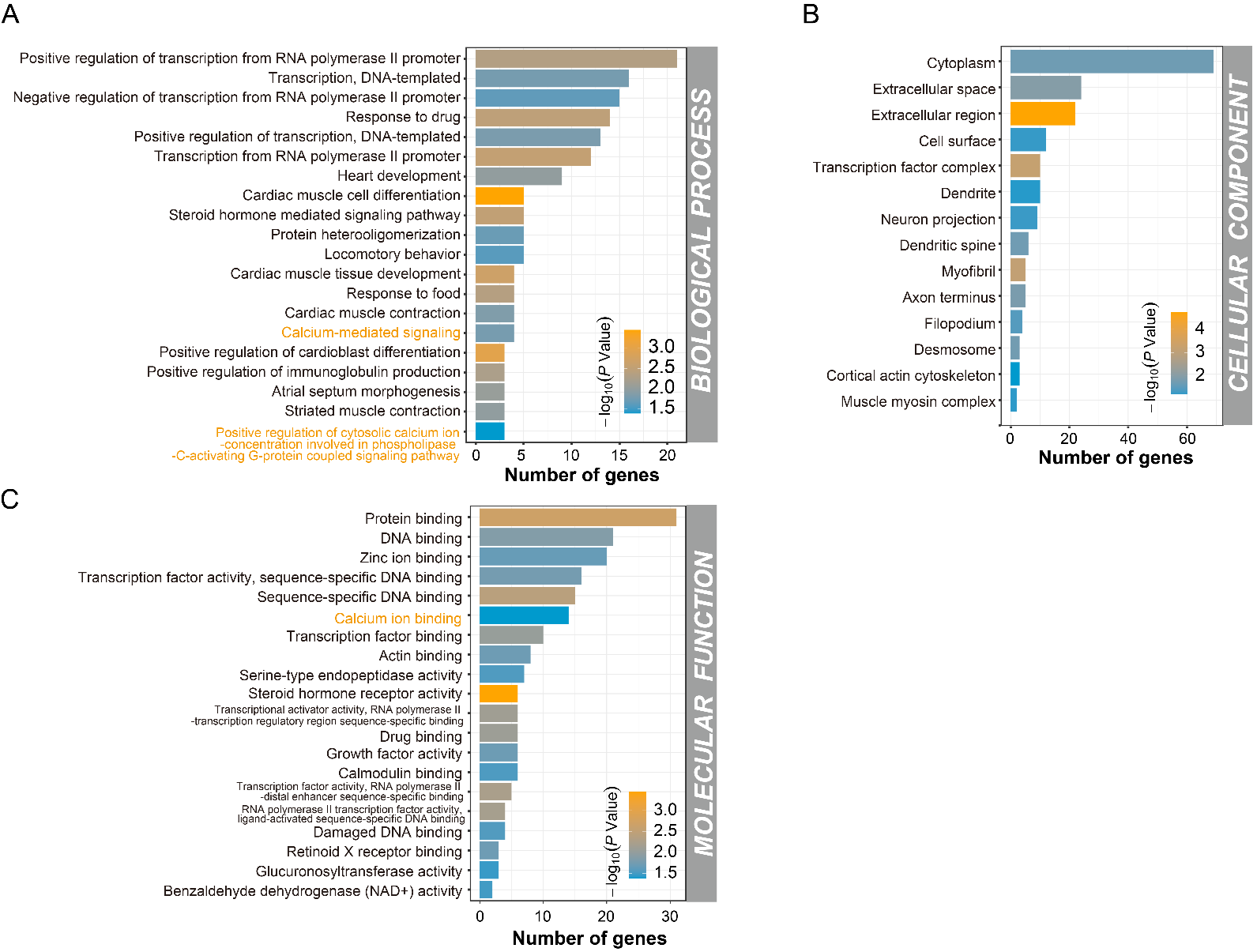
**Figure S3. Functional analyses of DEGs by GO classifications.** Functional annotation of the DEGs using GO terms of biological processes(A), cellular component(B), and molecular function(C). The enriched gene number is plotted as the abscissa and GO terms is plotted as the ordinate.

Fig.S4


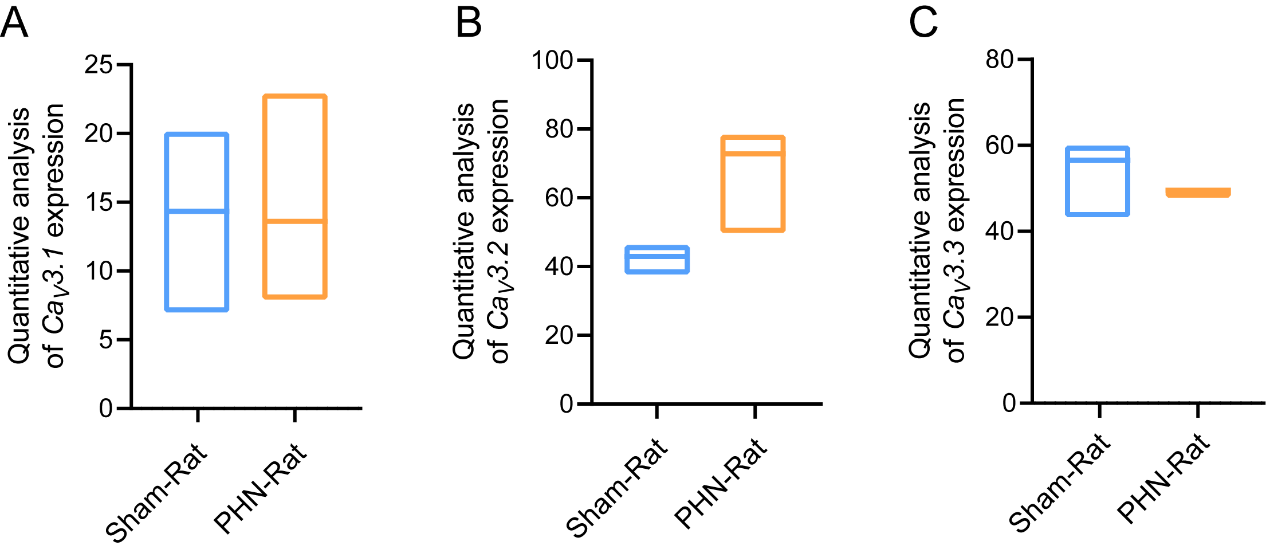


**Figure S4. Quantitative analysis of Ca_V_3.1, Ca_V_3.2, Ca_V_3.3 expression in dorsal root ganglion in sham and PHN rat.** The data comes from the GEO database (GSE64345).


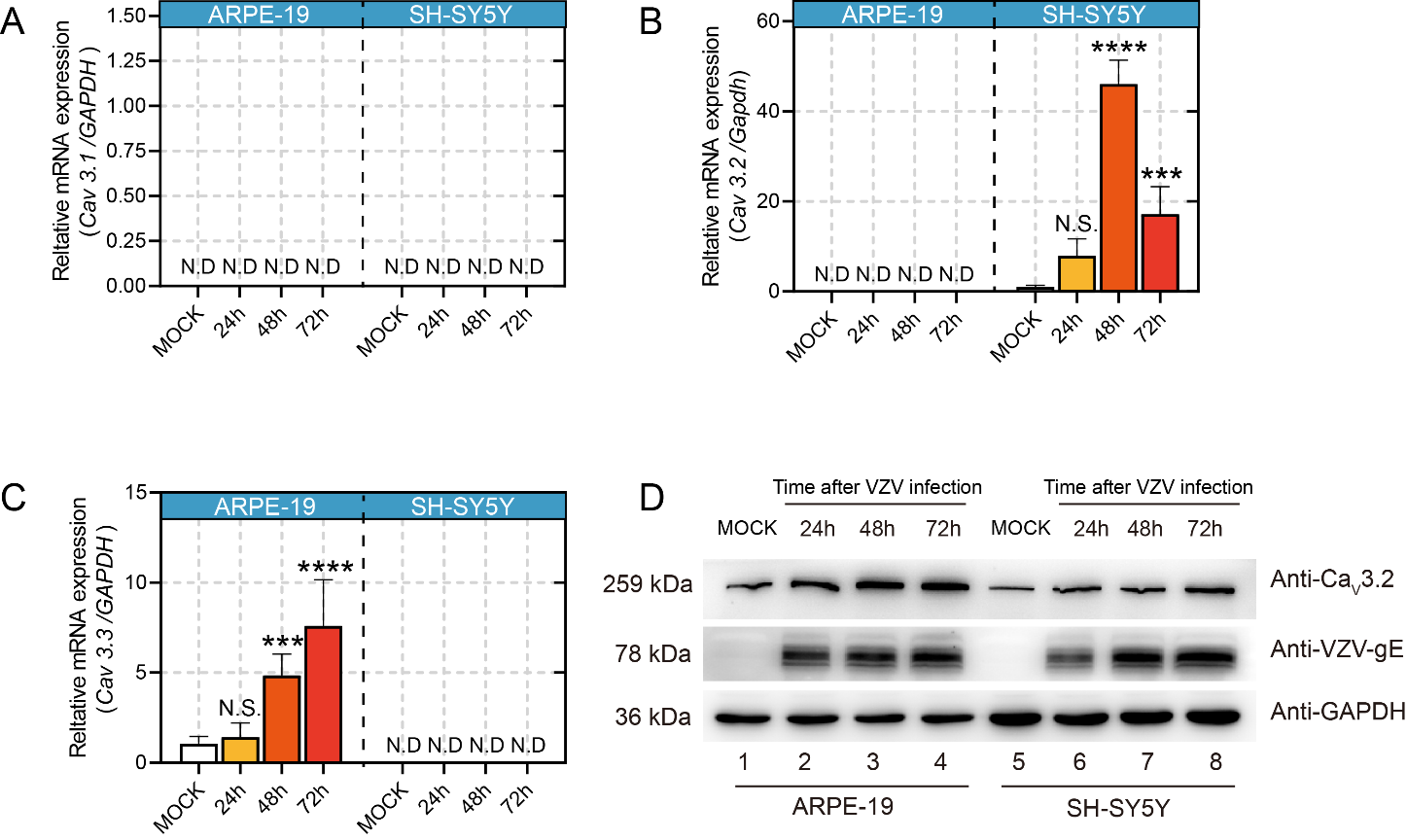
Fig.S5

**Figure S5. Expression of Ca_V_3.1, Ca_V_3.2, Ca_V_3.3 in ARPE-19 and SH-SY5Y cells in mock and VZV infection.** qRT-PCR results of *Ca_V_3.1*(A), *CaV3.2*(B) and *Ca_V_3.3*(C) mRNA expression in ARPE-19 and SH-SY5Y cells relative to *GAPDH* in mock and VZV infection. (D) Western blot results of Ca_V_3.2, VZV glycoprotein gE and GAPDH protein expression in ARPE-19 and SH-SY5Y cells in mock and VZV infection. ****P* < 0.001, *****P* < 0.0001, compared with MOCK group; N.S.: not significant.
